# Treatment Outcomes of Multidrug-Resistant Tuberculosis: A Systematic Review and Meta-Analysis

**DOI:** 10.1371/journal.pone.0006914

**Published:** 2009-09-09

**Authors:** James C. Johnston, Neal C. Shahidi, Mohsen Sadatsafavi, J. Mark Fitzgerald

**Affiliations:** 1 Tuberculosis Control, British Columbia Centre for Disease Control, Vancouver, Canada; 2 Centre for Clinical Epidemiology and Evaluation, Vancouver Coastal Health Research, Vancouver, Canada; 3 Collaboration for Outcome Research and Evaluation, University of British Columbia, Vancouver, Canada; McGill University, Canada

## Abstract

**Background:**

Treatment outcomes for multidrug-resistant *Mycobacterium Tuberculosis* (MDRTB) are generally poor compared to drug sensitive disease. We sought to estimate treatment outcomes and identify risk factors associated with poor outcomes in patients with MDRTB.

**Methodology/Principal Findings:**

We performed a systematic search (to December 2008) to identify trials describing outcomes of patients treated for MDRTB. We pooled appropriate data to estimate WHO-defined outcomes at the end of treatment and follow-up. Where appropriate, pooled covariates were analyzed to identify factors associated with worse outcomes. Among articles identified, 36 met our inclusion criteria, representing 31 treatment programmes from 21 countries. In a pooled analysis, 62% [95% CI 57–67] of patients had successful outcomes, while 13% [9]–[17] defaulted, 11% [9]–[13] died, and 2% [1]–[4] were transferred out. Factors associated with worse outcome included male gender 0.61 (OR for successful outcome) [0.46–0.82], alcohol abuse 0.49 [0.39–0.63], low BMI 0.41[0.23–0.72], smear positivity at diagnosis 0.53 [0.31–0.91], fluoroquinolone resistance 0.45 [0.22–0.91] and the presence of an XDR resistance pattern 0.57 [0.41–0.80]. Factors associated with successful outcome were surgical intervention 1.91 [1.44–2.53], no previous treatment 1.42 [1.05–1.94], and fluoroquinolone use 2.20 [1.19–4.09].

**Conclusions/Significance:**

We have identified several factors associated with poor outcomes where interventions may be targeted. In addition, we have identified high rates of default, which likely contributes to the development and spread of MDRTB.

## Introduction

Multidrug-Resistant Tuberculosis (MDRTB) refers to *Mycobacterium tuberculosis* (TB) strains with *in vitro* resistance to the two most effective anti-tuberculosis drugs, isoniazid (INH) and rifampin (RFP). MDRTB has become a major barrier to achieving successful control of TB, as therapy is less effective, associated with more adverse events and is more costly to treat when compared with standard first line therapy. According to a recent WHO report, approximately 490,000 MDRTB cases occur globally every year, corresponding to approximately 4.8% of the world's TB cases [1], [2]. The importance of addressing drug resistant TB is further amplified by more recent reports on extensively drug resistant TB (XDRTB) [3], which represented 7% of MDR isolates referred to supranational reference laboratories from 2000–2004 [1].

Inadequate treatment of MDRTB can lead to worse patient outcomes, while increasing the risk of extensive drug resistance [4]–[6]. Guidelines for the management of MDRTB have been developed over the past decade, but there is little evidence based on randomized controlled trials to support current recommendations [7], [8]. Moreover, treatment strategies have varied significantly and are difficult to compare between populations [8], [9]. This lack of evidence reflects a lack of political and financial will, in part from the perception that MDRTB is of limited epidemiological importance [7]. It also reflects the limited number of second line drugs that are available and the unequal distribution of access depending on local resources. The recent recognition of the increasing magnitude of MDRTB, along with the poor prognosis of XDRTB has created the impetus for a more evidence-based approach to the treatment of MDRTB.

Recently, standardized definitions were established to allow comparison between treatment groups and facilitate the development of a more evidence-based approach [9], [10]. We therefore decided to complete a systematic review of MDRTB treatment regimens. Where appropriate, we performed a meta-analysis to explore associations between MDRTB treatment outcomes and the clinical and microbiological factors that influence outcome. We aimed to identify all the published literature and to establish the best possible evidence base of clinical and microbiological predictors of treatment response.

## Methods

### Search strategy

Several search strategies were used to identify potentially relevant studies. Search strategy was developed by the investigators (Johnston and Shahidi) with consultation of a medical librarian.

1. A systematic search was conducted to identify eligible studies in the following databases: EMBASE (1980 to Week 50, 2008), MEDLINE (1965 to Week 50, 2008), International Pharmaceutical Abstracts (1970 to November 2008) and BIOSIS (1969 to Week 50, 2008). Keywords included tuberculosis, TB, multi$, drug$, multidrug, multi-drug, MDRTB, MDR TB, MDR-TB, extensively drug resistant, extensively drug-resistant, XDRTB, XDR TB, XDR-TB, drug resistant tuberculosis.
2. Key word search was conducted in EBM Reviews - Cochrane Database of Systematic Review, Database of Abstracts of Reviews of Effects, and Cochrane Central Register of Controlled Trials (all to 4th Quarter 2008). Citations were reviewed, revealing no systematic reviews on this subject.
3. Keyword and title search of Web of Science was performed using the terms tuberculosis, TB, tb, multiple drug resistant, multiple drug resistance, multi-drug resistance, multi-drug resistant, multi-drug-resistant, drug resistant, MDR, MDRTB, extensively drug resistant, XDRTB.
4. Hand searching of the following journals: *International Journal* of Tuberculosis and Lung Disease, *Chest*, *American Journal of Respiratory & Critical Care Medicine*, and *Clinical Infectious Disease*.
5. Bibliographies of full text articles were examined for eligible studies.
6. Results were limited to English articles. Abstracts were included in search results.

### Selection of Studies

Studies obtained from the literature search were checked by title and citation. If an article appeared relevant, the abstract was reviewed. Relevant abstracts were examined in full text. Inclusion criteria were as follows: an original study; reported in English; reported treatment outcomes in a population of adult, culture-confirmed MDRTB patients; reported outcomes presented in a format allowing for comparison with other studies. Exclusion criteria were as follows: exclusive surgical series; exclusive use of first-line therapy in the treatment protocol.

### Validity assessment

Studies were assessed for quality, with only high quality studies included for analysis. High quality studies reported outcomes on at least 10 patients; were prospective cohort, retrospective consecutive cohort, consecutive case control or randomized control in design; reported an average treatment duration of ≥12 months within an average follow-up duration ≥18 months; reported basic demographic data; reported less than 1/3 default or lost to follow-up. When study populations overlapped, we included the more recent and larger study population in the analysis. If the smaller population provided data on an outcome or variable not reported in the larger study, results were included for that specific variable.

### Outcome measures

Measured outcomes reflect the definitions proposed by *Laserson et al.*, and published in recent WHO guidelines [9], [10]. Successful outcomes included patients meeting the definition of *Cure* or *Treatment Completed*. Unsuccessful outcomes included patients meeting the definition of *Death, Defaulted*, *Failed* or *Transferred Out*. When follow-up data was used, relapse was included as an unsuccessful outcome. To homogenize data, end-of-treatment (EOT) and follow-up (FUP) outcomes were separated for analysis. FUP outcomes refer to post-treatment follow-up, with follow-up duration measured in months. If studies were unable to meet WHO definitions, reviewers established outcomes to reflect these definitions. Certain studies reported data that precluded the use of WHO outcome definitions, and were not included for analysis. Several variables–including co-morbidities, demographic variables, microbiological profile, disease presentation and disease characteristics—were collected for outcome analysis. Analysis of these variables pooled both EOT and FUP outcomes given the paucity of homogenous data for individual variables. Where appropriate we contacted investigators for additional data as well as for clarification of their findings.

### Data extraction and statistical analysis

Data abstraction was performed by two reviewers using a standardized abstraction form. When there was disagreement, the relevant paper was reviewed and differences were resolved by consensus. Microsoft Excel (version 12.0), STATSDirect version 2.7.6 (StatsDirect Ltd, Cheshire, UK), and STATA version 10.0 (STATA Corporation, College Station, Texas) software were used for data entry and analysis. Study characteristics and treatment outcomes were summarized in tables. Data related to treatment outcomes were pooled from published studies as described above. We pooled the proportion of successful outcome, death, default, transfer of care, and failure across studies using a random-effects meta-analysis. Heterogeneity of studies was estimated by calculating I^2^ and Cochrane p values. The heterogeneity of binary covariates was estimated through p values. The overall effect of the dichotomous data was carried out by using a random effects analysis because of study heterogeneity (I^2^ >50%, p<0.05) and measured by odds ratio with 95% confidence intervals [95%CI].

## Results

**Figure 1:**
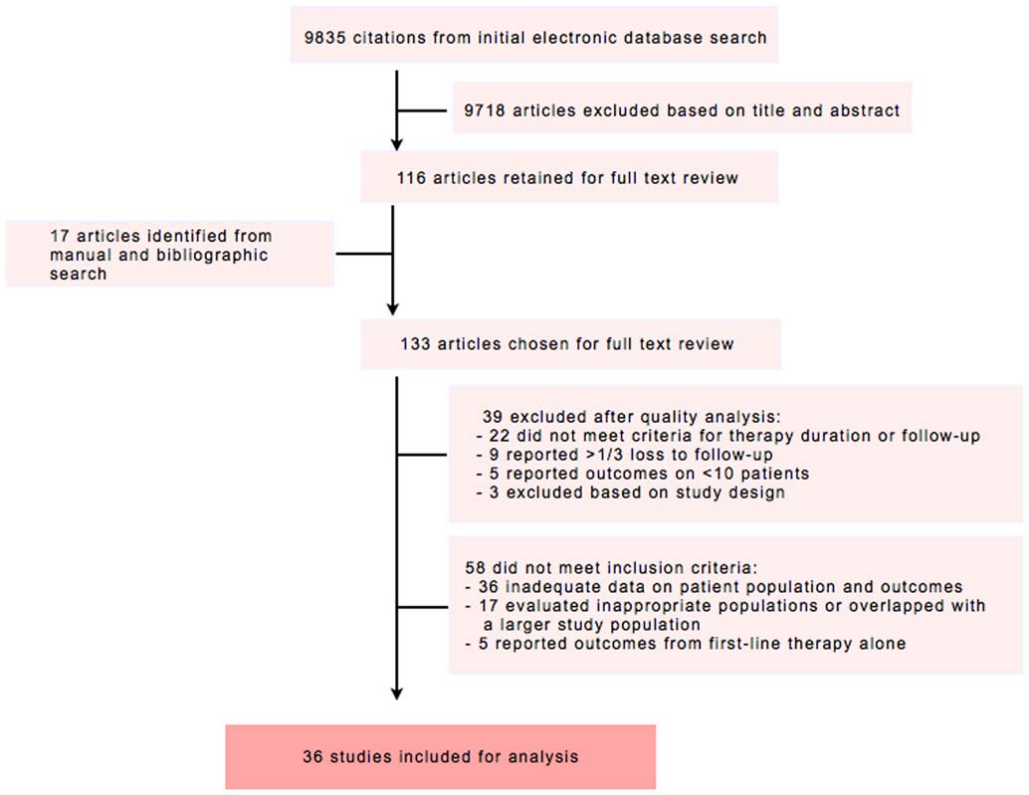
Forest plots of covariates.

We identified 9835 citations from our initial electronic database search. Of these, 116 articles were identified for full text review. A further 17 articles were identified through manual search, bibliographic search, and reviewer suggestions. Of the 133 articles reviewed in full text, 36 articles, representing 31 distinct patient populations were analyzed based on our *a priori* inclusion-exclusion criteria and quality assessment [11]–[46]. Ninety-four studies were removed prior to analysis: 36 lacked adequate data on the patient population or outcomes; 17 evaluated an inappropriate population or overlapped with a larger study population; 5 reported outcomes based on first line therapy alone; 22 did not meet criteria for treatment duration or follow-up; 9 reported more than one-third default or loss to follow-up; 5 reported outcomes on <10 patients; and 3 were excluded based on study design (Figure 1).

The 36 articles chosen for review reported treatment outcomes on 31 study populations from 21 countries on 5 continents (Table 1). The study periods ranged from 1973 to 2006. The majority were retrospective chart reviews, with five prospective cohorts [17], [28], [37], [42], [43] and one retrospective case control [32]. The average study population (range) was 210 (24–1407). The majority of patients were male, with a mean of 68% (48–92) across all studies. In 30 studies with available data, the mean age was 40 years. All studies reported on patients with a prior history of treatment. In studies with available data, 84% (32–100) of patients had previously received anti-tuberculous therapy. The average number of drugs to which resistance was shown was 4.1 for studies reporting resistance. For those reporting first and second-line resistance, the average number of drugs to which there was resistance was 4.4.

**Table 1:** Summary of studies.

Study Period	Design	Sample Size	Region	Endpoint	Male (%)	HIV (%)	DM (%)	ETOH (%)	BMI (%)	SMR (%)	CAV (%)	SRG (%)	PRT (%)	6DR (%)	FQU (%)	XDR (%)	Covariates Analyzed
1986–1999	RC	40	Canada	33M FUP	53	0	-	-	-	-	-	-	-	-	-	-	-
1993–2006	RC	540	USA	EOT	-	7	-	-	-	-	-	-	-	-	-	3	XDR
2001–2004	RC	45	Czech Republic	EOT	82	0	-	-	-	-	-	-	-	-	-	-	-
1982–2000	RC	48	USA	EOT, 24M FUP	67	23	-	-	-	-	-	6	-	-	-	-	HIV, SRG
1992–1996	RC	299	Taiwan	EOT, 39M FUP	72	0	-	-	-	92	-	-	94	-	42	-	GEN, SMR, PRT, FQU
2003–2005	RC	87	Uzbekistan	EOT	61	-	-	23	-	-	-	-	-	-	-	-	ETOH
1998–2000	PC	25	Spain	EOT, 24M FUP	92	0	-	-	-	-	-	8	88	32	72	-	SRG, PRT, 6DR, FQU
1995–1999	RC	127	Italy	EOT	65	12	9	5	-	87	-	-	-	-	58	-	GEN, HIV, DM, ETOH, SMR, FQU
1973–1983	RC	134	USA	51M FUP	69	-	-	-	-	87	-	-	-	55	-	-	GEN, SMR, 6DR
1994–2005	RC	407	USA	EOT	59	-	-	-	-	-	-	-	-	-	-	-	-
1989–1998	RC	24	Canada	EOT	75	4	-	-	-	-	-	-	-	-	-	-	-
2000	RC	167	Latvia	EOT	78	-	-	-	-	-	-	-	24	-	-	-	PRT
2000–2004	RC	608	Russian Fed	EOT	83	1	-	43	43	-	-	9	-	-	-	5	GEN, HIV, ETOH, BMI, SRG, XDR
1996–2005	RC	211	Korea	EOT	61	0	14	-	37	-	76	30	29	-	-	20	GEN, DM, BMI, CAV, SRG, PRT, XDR
2000–2002	RC	1407	Korea	EOT	74	<1	-	-	13	-	43	4	-	-	-	5	GEN, BMI, CAV, SRG, XDR
1998–2004	RC	155	Korea	EOT	53	0	15	1	28	86	71	23	10	-	-	17	GEN, DM, ETOH, BMI, SMR, CAV, SRG, PRT, XDR
2000	RC	204	Latvia	EOT	77	1	6	62	19	-	78	10	19	-	-	-	GEN, DM, ETOH, BMI, CAV, SRG, PRT
2002–2006	PC	43	Iran	EOT	63	0	-	-	-	-	-	-	-	-	-	-	GEN
1996–1999	RC	75	Peru	EOT, 40M FUP	49	1	-	-	43	-	-	-	-	-	-	-	GEN, BMI
1999–2002	RC	646	Peru	25M FUP	60	1	-	-	-	-	-	15	-	90	-	7	SRG, 6DR, XDR
1994–1997	RC	81	USA	EOT	67	38	-	-	-	-	-	-	-	-	-	-	-
1982–2004	CC	42	United Kingdom	EOT	50	0	-	-	-	-	-	-	-	-	-	-	-
1996–2002	RC	141	Argentina	EOT	48	0	-	-	-	-	-	6	-	-	-	-	SRG
1998–2000	RC	142	Korea	EOT	79	0	-	-	-	-	-	-	-	33	26	-	GEN, 6DR, FQR
1992–2002	RC	491	South Africa	24M FUP	59	3	-	29	-	-	-	6	91	-	-	-	GEN, HIV, ETOH, SRG, PRT
2000–2002	RC	244	Russian Fed	EOT	87	0	-	-	-	-	-	12	-	7	-	-	SRG, 6DR
1997–1999	PC	298	Peru	EOT	57	-	-	-	-	-	-	-	-	-	-	-	-
1992–1999	RC	158	Turkey	EOT	87	-	-	-	-	-	-	-	-	-	-	-	-
1991–1994	RC	25	USA	EOT	56	0	-	-	-	-	-	-	-	-	-	-	-
1992–2004	RC	252	Turkey	EOT	81	-	-	-	-	-	-	26	-	-	85	-	GEN, SRG, FQU
1999–2002	RC	118	Phillipines	EOT	86	-	-	-	-	-	-	-	96	-	-	-	PRT
1998–1999	PC	45	France	EOT	53	20	-	-	-	-	-	-	-	-	-	-	-
1997–1999	RC	58	Bangladesh	EOT, 24M FUP	84	-	-	-	-	-	-	-	-	-	-	-	-
1989–2000	RC	44	Vietnam	EOT, 15M FUP	59	0		-	-	-	-	-	-	-	-	-	-
1990–1997	RC	72	Hong Kong	EOT, 25M FUP	74	0	-	-	-	90	51	-	-	-	85	-	GEN, SMR, CAV, FQR
1990–2000	RC	72	Hong Kong	EOT	74	0	-	-	-	-	-	-	-	-	40	-	FQU

RC = Retrospective cohort, PC = prospective cohort, CC = case control, GEN = gender, HIV = HIV positive, DM = diabetes, ALC = alcohol abuse, BMI = low body mass index, SMR = smear positive, CAV = cavitary disease, PRT = prior therapy, 6DR = six drug resistance, FQR = fluoroquinol.

In the 26 trials with a total 4959 patients reporting EOT outcomes 62% [95% CI 57–67] of patients met the definition of successful treatment, while 11% [9]–[13] of patients died and 8% [5]–[11] failed therapy (Table 2). The default rate was 13% [9]–[17], while 2% [1]–[4] had their care transferred to another jurisdiction. In 9 trials with a total of 1583 patients reporting outcomes at follow-up, the mean follow-up duration was 27 [15]–[51] months (Table 3). The percentage with successful outcomes at follow-up was 64% [56–72], while 14% [10]–[19] died, 13% [7]–[20] defaulted, 5% [2]–[9] failed therapy, 1% [0–2] had their care transferred and 2 [0–4] relapsed. Based on the data provided, the percentage of patients presenting with re-infection, rather than relapse cannot be determined.

**Table 2:** End of Treatment Outcomes.

Author (Reference)	Population	Successful Outcome	Death	Default	Transfer of Care	Failure
Bartu [13]	45	33	11	1	0	0
Chiang [15]	299	153	28	87	0	31
Cox [16]	87	54	13	12	0	8
Escudero [17]	25	21	0	4	0	0
Ferrara [18]	126	49	11	21	16	29
Granich [20]	338	231	49	10	48	0
Hersi [21]	18	8	5	1	4	0
Keshavjee [23]	608	400	31	119	0	58
Kim HR [24]	211	132	19	7	7	46
Kim DH [25]	1407	637	144	453	108	65
Kwon [26]	155	102	10	15	6	22
Leimane [27]	204	135	14	26	0	29
Masjedi [28]	43	29	8	0	0	6
Mitnick 2003 [30]	75	55	5	14	0	1
Narita [31]	81	46	26	9	0	0
O'Riordan [32]	42	25	3	9	4	1
Palmero [33]	141	73	27	28	0	13
Park [34]	142	63	4	41	15	19
Suarez [37]	298	136	32	34	0	96
Telzak [39]	25	16	1	0	0	8
Torun [40]	252	193	18	25	0	16
Tupasi [41]	118	71	18	16	1	12
Uffredi [42]	45	26	12	7	0	0
Van Deun [43]	58	40	8	7	0	3
Ward [44]	44	38	2	3	0	1
Yew [45]	72	51	6	6	0	9
Summary	191	62%	11%	13%	2%	8%
CI		[57–67]	[9]–[13]	[9]–[17]	[1]–[4]	[5]–[11]
I^2^		91%	80%	94%	93%	94%
P value		<0.0001	<0.0001	<0.0001	<0.0001	<0.0001

**Table 3:** Outcomes at Follow-up.

Author (Reference)	Months FUP	Sample Size	Successful Outcome	Death	Relapse	Transfer of Care	Failure	Default
Avendano [11]	33	40	28	5	4	0	1	2
Burgos [14]	24	48	31	11	2	3	0	1
Escudero [16]	24	25	21	0	0	0	0	4
Goble [18]	51	159	78	27	0	0	32	22
Mitnick 08 [29]	25	646	412	134	17	0	18	65
Shean [34]	24	491	239	68	0	10	30	144
Van Deun [42]	24	58	35	11	2	0	3	7
Ward [43]	15	44	38	2	0	0	1	3
Yew [44]	25	72	46	8	1	0	4	13
Summary	27	176	64%	14%	2%	1%	5%	13%
CI			[56–72]	[10]–[19]	[0–4]	[0–2]	[2]–[9]	[7–20
I^2^			87%	74%	80%	70%	85%	91%
p value			<0.0001	0.0002	<0.0001	0.0009	<0.0001	<0.0001

### Covariate analysis

**Figure 2:**
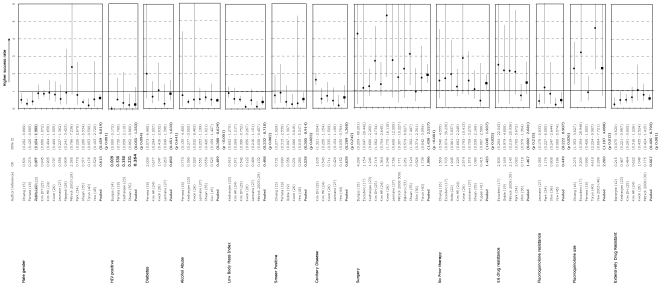
Summary of literature search and study selection.

Predetermined covariates (gender, diabetes, alcohol abuse, HIV positive, BMI, smear positive, cavitary disease, surgical intervention, prior treatment, ≥6 drug resistance, fluoroquinolone use, and presence of XDRTB) were pooled and analyzed. The following covariates were associated with poor outcome: male gender (pooled OR for successful outcome 0.61, [95% CI0.46–0.82]), alcohol abuse 0.49 [0.39–0.63], low BMI 0.41 [0.23–0.72], smear positivity 0.53 [0.31–0.91], fluoroquinolone resistance 0.45 [0.22–0.91], and XDR resistance pattern 0.57 [0.41–0.80]. Surgical intervention 1.91 [1.44–2.53], no prior TB treatment 1.42 [1.05–1.94] and fluoroquinolone use 2.20 [1.19–4.09] were associated with successful outcome. Diabetes, cavitary disease, HIV and resistance to ≥6 drug were not statistically associated with worse outcomes. Two covariates, fluoroquinolone use and XDRTB, were analyzed for an association with death. The use of fluoroquinolones was associated with decreased mortality 0.30 [0.15–0.61], while there was a trend towards increased mortality with XDR (Figure 2).

## Discussion

Drug resistant TB ultimately develops from the inadequate treatment of active pulmonary TB. There are multiple reasons for inadequate therapy; poor prescribing practices with insufficient treatment duration and poor drug selection are well-recognized contributors [5], [47]. Systemic problems, through inadequate public health resources and unpredictable drug supplies also play a role [4]. In addition, irregular medication intake–whether from insufficient patient education, adverse events, or socioeconomic determinants–contribute to resistance. There are also a significant proportion of patients who acquire drug resistant disease because they live in an environment with a high prevalence of drug resistant disease.

Despite these well-known causes of drug resistance, our review of 36 observational studies revealed very high rates of default and transfer of care. The majority of unsuccessful outcomes at end of treatment were the result of these factors, with relapse accounting for only 2% of patients in follow-up. Moreover, default appears to be a global phenomenon, with rates over 15% in several countries, including Korea (32%) [25], Taiwan (29%) [15], Russia (20%) [23], Italy (17%) [18], Spain (16%) [17], South Africa (29%) [35], Argentina (20%) [33], and Peru (19%) [29]. With the advent of DOTS and DOTS-Plus strategies, MDRTB rates have improved in some high prevalence countries, such as Latvia where new MDR cases have decreased more than two-fold between 2000 and 2007 (V. Leimane, unpublished data). Yet under these improved circumstances, published default rates were above 10% [27].

The consequences of inadequate therapy are also apparent in our data. On pooled analysis we have shown that prior treatment, fluoroquinolone resistance, and XDRTB were all associated with poor outcomes. While resistance to fluoroquinolones was associated with worse outcomes, fluoroquinolone use was associated with successful outcomes. This finding is consistent with several observational studies showing that fluoroquinolones are highly effective in MDRTB treatment [15], [38], [40], [45], [46], [48], [49]. The WHO Green Light Committee recommends that MDR patients receive a fluoroquinolone when their culture results reveal a susceptible isolate [10]. But judicious use of fluoroquinolones is crucial in suspected and confirmed cases of TB if we hope to impede the development of further resistance [29]. What is less clear is the role of newer generation fluoroquinolones in the treatment of fluoroquinolone resistant MDRTB. The findings of Yew suggest that newer generation fluoroquinolones may be effective against ofloxacin resistant strains [46]. Further studies will be necessary to clarify the role of fluoroquinolones in this population. The emerging XDRTB literature may be a resource to explore this issue.

Since the seminal report on XDRTB from KwaZulu-Natal, South Africa [3], multiple studies have reported outcomes of XDRTB treatment [12], [23]–[26], [30], [50]–[53]. In the XDR cohorts examined in our study, the majority of patients had poor outcomes, despite low HIV rates. The global burden of XDRTB reflects the multiple deficiencies of tuberculosis control [47]. With aggressive, comprehensive individualized therapy, programs can improve XDRTB outcomes [30]. But, targets for interventions need to be better defined.

An obvious target for comprehensive interventions is in the management of co-morbidities. The most commonly reported co-morbidities in our selected studies were alcohol abuse, low BMI, and diabetes. The association between poor outcome with BMI and alcohol abuse is not unexpected. Body mass index, as a symptom of severe disease and low socioeconomic status requires aggressive intervention [29]. To our knowledge, there are no comparative studies examining the optimal nutritional intervention in an MDRTB population. Moreover, there are no evidence-based guidelines on nutritional supplementation in adults or children [54]. Further studies on MDRTB could not only include a baseline nutritional intervention, but also study the impact of various nutritional interventions on MDRTB outcomes. Meanwhile, alcohol abuse has been associated with poor TB outcome in several studies [16], [23], [35], [36], [55] and measures to improve care for alcohol abuse should be undertaken. A recently published study in Tomsk, Russia, describes successful adoption of a program to address alcohol use disorders, with the intent to measure outcomes [56].

Diabetes mellitus is also a well-described co-morbidity in MDRTB patients. Not only are diabetics prone to reactivation TB [57], they may also be at increased risk of developing MDRTB [58], [59]. The reasons for this are unclear, but based on our pooled data, diabetics do not appear to have worse outcomes with MDRTB. These results stand in contrast to previously published literature on drug sensitive TB [60], and may relate to our small sample size of 68 diabetics.

Lastly, our data supports the role of surgery in the management of MDRTB. Surgical patients appear to have superior outcomes—this may reflect the practice of performing surgery on less sick, adherent patients who have responded favourably to initial medical therapy. Although trials in surgery are inherently difficult, they should be undertaken to examine the role of surgery in MDRTB patients. Multiple surgical series have been published on this topic and could be examined for predictors of successful outcomes. In addition, future trials examining the role of surgery on highly resistant strains such as fluoroquinolone and XDR strains may be valuable.

Our study has several limitations. The most important is our inability to identify any randomized control trials (RCTs). Instead we relied exclusively on observational data for treatment outcomes. This likely introduced confounding to our pooled analysis, since crude outcomes, rather than adjusted odds ratios were reported for most trials. Moreover, the analysis of outcomes and pre-determined covariates used heterogeneous data from variable treatment regimens. The trends introduced by this analysis are subject to bias and should be interpreted with caution. Our quality analysis also likely introduced bias since only systems with resources for record keeping, second-line therapy and prolonged treatment and follow-up were included for analysis. These systems likely have improved outcomes compared with the many poorly resourced TB control programs throughout the world. Indeed, a recent editorial noted an “exceptionality” bias when commenting on reported XDRTB outcomes [61].

Of note, our treatment outcome definitions were heterogeneous between populations. Of the 36 studies included for analysis, only 12 studies (representing nine patient populations) met the outcome definitions proposed in WHO guidelines [10], [16], [22]–[27], [30], [35], [36], [40], [41]. The effect of this heterogeneity is difficult to assess. We observed, however, that several studies did not use the stringent culture-based outcome definitions such as *Cure* or *Failed*. This likely biased results towards more successful outcomes. Despite this, studies that used WHO outcomes showed similar outcomes: 62% [5–69] of patients had successful outcomes with a 15% [9]–[22] default and 10% [7]–[14] death rate.

Another significant limitation is the lack of data on HIV positive patients. In total 143 of 6359 patients (2%) were known to be HIV positive, with the data from 39 patients available for outcome analysis. This limits the power to detect the effect of the presence of HIV infection. Some studies reported HIV infected populations with treatment and follow-up durations shorter than 12 and 18 months respectively [3], [62], [63]. Thus, our inclusion criteria for treatment duration and follow-up likely excluded a disproportionate population of HIV positive patients from our analysis. In addition, our criteria eliminated studies examining ‘outbreaks’ of MDRTB, where a more virulent strain spreads quickly through a community. Such outbreak investigations tend to have limited follow-up and outcome data given the urgency to report findings [64], [65].

Recently, a systematic review examining MDRTB treatment outcomes in 33 populations was published by *Orenstein et al*. [66]. In this review, 62% of patients were classified as successful outcomes. Covariate analysis revealed that the proportion of patients with successful treatment outcomes did not significantly change for several individual covariates. The authors concluded that this likely reflected study heterogeneity. Similarly, our review examined MDRTB treatment outcomes. We have, however, focused on underlying patient and disease characteristics–such as diabetes, alcoholism, and smear positivity—while, *Orenstein et al*. focused on programmatic characteristics such as length of treatment and the use of Directly Observed Therapy. Overall, our outcomes do not differ markedly from those of *Orenstein et al.*, and we feel that our covariate analysis contributes to the literature on this topic.

Though treatment success remains dismally low in MDRTB populations, there is reason to be hopeful. With the advent of standardized nomenclature, outcomes of MDRTB treatment regimens can be evaluated with more precision. The addition of more comprehensive therapeutic strategies will also help improve outcomes. But, an evidence-based approach, in addition to comprehensive, individualized therapy, is necessary to contain MDRTB [7]. Without high quality evidence to support therapeutic decisions, including data from high-risk populations such as HIV positive and diabetic patients, high mortality, default, and failure rates will persist.
